# Decreased fucosylated PSA as a urinary marker for high Gleason score prostate cancer

**DOI:** 10.18632/oncotarget.10987

**Published:** 2016-08-01

**Authors:** Kazutoshi Fujita, Takuji Hayashi, Kyosuke Matsuzaki, Wataru Nakata, Mika Masuda, Atsunari Kawashima, Takeshi Ujike, Akira Nagahara, Mutsumi Tsuchiya, Yuka Kobayashi, Satoshi Nojima, Motohide Uemura, Eiichi Morii, Eiji Miyoshi, Norio Nonomura

**Affiliations:** ^1^ Department of Urology, Osaka University Graduate School of Medicine, Suita, Japan; ^2^ Department of Molecular Biochemistry and Clinical Investigation, Osaka University Graduate School of Medicine, Suita, Japan; ^3^ J-Oil Mills, Inc., Yokohama, Japan; ^4^ Department of Pathology, Osaka University Graduate School of Medicine, Suita, Japan

**Keywords:** prostate cancer, fucosylation, PSA, urine, Gleason score

## Abstract

Fucosylation is an important oligosaccharide modification associated with cancer and inflammation. We investigated whether urinary fucosylated PSA (Fuc-PSA) levels could be used for the detection of high Gleason score prostate cancer. Urine samples were collected from men with abnormal digital rectal examination findings or elevated serum PSA levels, before prostate biopsy. Lectin-antibody ELISA was used to quantify the Lewis-type or core-type fucosylated PSA (PSA-AAL) and core-type fucosylated PSA (PSA-PhoSL) in the urine samples. Both types of urinary Fuc-PSA were significantly decreased in the men with prostate cancer compared with the men whose biopsies were negative for cancer (*P* = 0.026 and *P* < 0.001, respectively). Both were also significantly associated with the Gleason scores of the biopsy specimens (*P* = 0.001 and *P* < 0.001, respectively). Multivariate analysis showed that PSA density, urinary PSA-AAL, and urinary PSA-PhoSL were independent predictors of high Gleason score prostate cancer. The area under the receiver-operator characteristic curve (AUC) value for the prediction of cancers of Gleason score ≥ 7 was 0.69 for urinary PSA-AAL and 0.72 for urinary PSA-PhoSL. In contrast, the AUC value was 0.59 for serum PSA, 0.63 for PSA density, and 0.58 for urinary PSA. In conclusion, a decreased urinary Fuc-PSA level is a potential marker for the detection of high Gleason score prostate cancer.

## INTRODUCTION

An elevated prostate-specific antigen (PSA) level and abnormal digital rectal examination (DRE) finding will prompt the clinician to perform a prostate needle biopsy for the diagnosis of prostate cancer. However, up to 40% of patients newly diagnosed with prostate cancer are categorized as low risk [1], in that they have a very limited possibility of disease progression and do not require definitive therapy. Because the PSA test cannot predict high Gleason prostate cancer, the US Preventive Services Task Force does not recommend PSA-based tests for screening this disease. Therefore, the search for a new marker associated with the high risk for prostate cancer is necessary.

Fucosylation is an important oligosaccharide modification associated with cancer and inflammation [2]. Three types of fucosylation have been identified; namely, α1-2 fucosylation (H type), α1-3/α1-4 fucosylation (Lewis type), and α1-6 fucosylation (core type) [2–5]. We have previously reported a high expression of serum fucosylated haptoglobin (which is associated with the GS) and α1-6 fucosyltransferase in prostate cancer cells [6]. PSAs have one N-glycosylation site, and 70% of the molecules contained a fucose group [7]. These findings led us to hypothesize that fucosylated PSAs (Fuc-PSAs) might be a predictor of prostate cancer, especially of the high-risk type.

Urine is a promising source of new biomarkers for prostate cancer, and urinary markers such as PCA3 and the TMPRSS2-fusion gene have been reported [8]. In this study, we developed a lectin-antibody ELISA for the detection of Lewis-type or core-type Fuc-PSA (which binds with *Aleuria aurantia* lectin; PSA-AAL) and core-type Fuc-PSA (which binds with *Pholiota squarrosa* lectin; PSA-PhoSL). We also tested whether the Fuc-PSA level in urine, after DRE, could specifically detect aggressive prostate cancer.

## RESULTS

### Association of urinary fucosylated PSA levels with the Gleason score

**Figure 1:**
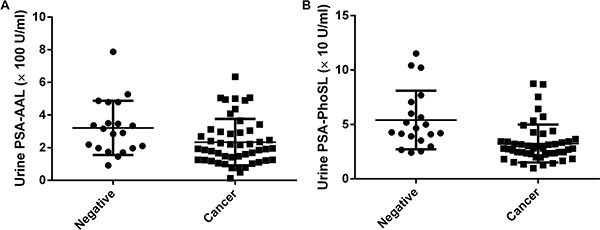
Urinary fucosylated PSA levels were associated with results of the prostate biopsy (**A**) Urinary PSA-AAL (*P* = 0.0268); (**B**) Urinary PSA-PhoSL (*P* = 0.0001).

**Figure 2:**
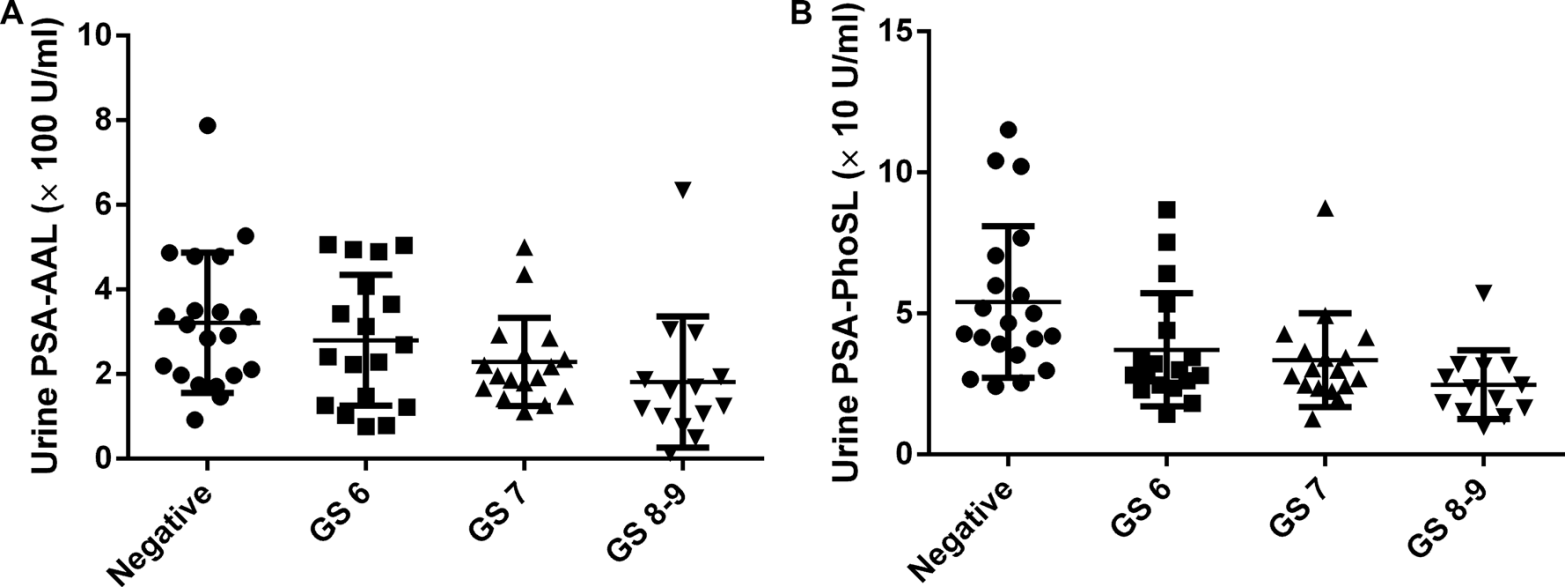
Urinary fucosylated PSA levels were associated with the Gleason score of biopsy specimens (**A**) Urinary PSA-AAL; (**B**) Urinary PSA-PhoSL.

Table 1 showed the clinicopathological characteristics of samples from donors who underwent a prostate needle biopsy. There were no associations between the GS and patient characteristics. As mentioned above, AAL binds specifically to Lewis-type and core-type fucosylation sites whereas PhoSL binds only to core-type fucosylation sites. The urinary PSA-AAL and PSA-PhoSL levels were significantly higher in patients with negative biopsy than in the patients with prostate cancer (*P* = 0.026 and *P* = 0.0001, respectively) (Figure 1). Urinary PSA-AAL was significantly correlated with urinary PSA-PhoSL (Spearman's *r* = 0.477, *P* < 0.001), whereas urinary PSA-PhoSL was significantly correlated with urinary PSA (Spearman's *r* = 0.273, *P* = 0.026). When patients were categorized into four groups (viz., negative, GS 6, GS 7, and GS 8–9), the decreased urinary PSA-AAL and PSA-PhoSL levels were significantly associated with a higher GS (Jonckheere-Terpstra test for trend; *P* = 0.001 and *P* = 0.0001, respectively) (Figure 2). Urinary PSA-AAL was not correlated with the percentage of positive cores (Spearman's *r* = −0.178, *P* = 0.240) or the percentage of prostate cancer core length (Spearman's *r* = −0.195, *P* = 0.199), and the same were true for urinary PSA-PhoSL (percentage of positive cores: Spearman's *r* = −0.167, *P* = 0.277; percentage of prostate cancer core length: Spearman's *r* = −0.093, *P* = 0.545).

**Table 1:** Patient characteristics and results

	Overall	Biopsy specimen				
		Negative	GS 6	GS 7	GS 8-9	*p* for trend
Number	69	20	18	17	14	
Age (year)	69 (56–83)	66.5 (56–80)	68 (56–80)	70 (61–77)	68.5 (59–83)	0.21
Serum PSA (ng/ml)	7.80 (2.99–26)	8.32 (3.96–19.79)	6.75 (2.99–21.7)	8.9 (5.08–26.0)	7.77 (4.33–18.2)	0.33
Prostate volume (ml)	29.8 (9.2–90)	33.6 (9.2–70.0)	29.4 (20.0–90.0)	29.1 (10.0–86.0)	27.8 (14.0–67.5)	0.44
PSA density	0.25 (0.08–2.6)	0.22 (0.09–0.67)	0.25 (0.08–0.75)	0.36 (0.19–2.60)	0.26 (0.10–0.97)	0.10
Urine PSA (ng/ml)	70.1 (0.35–194)	93.6 (4.5–179.5)	71.2 (0.35–188.7)	87.1 (25.1–194.2)	47.4 (1.30– 100.4)	0.14

### Prediction of Gleason score by urinary PSA-AAL and PSA-PhoSL levels

**Figure 3:**
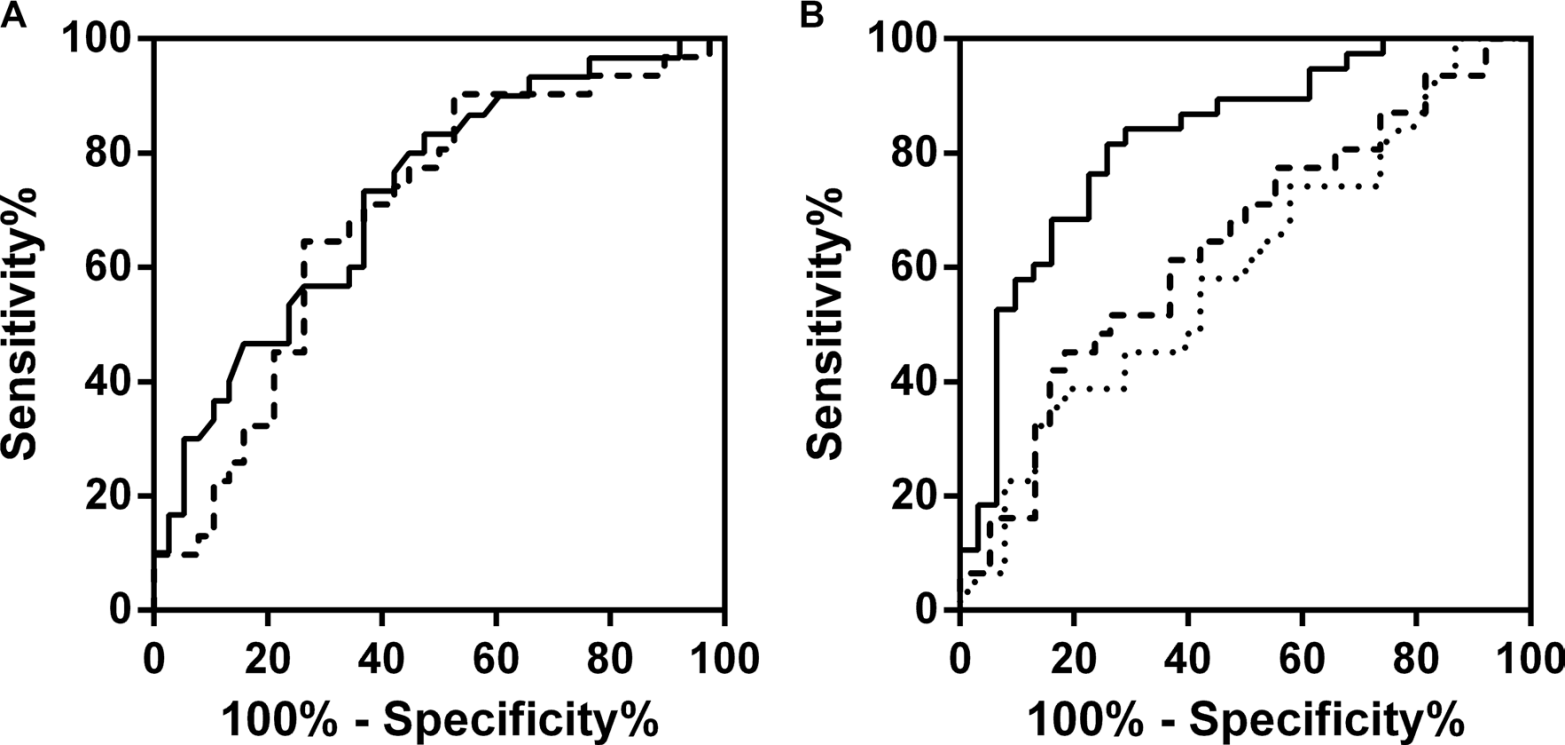
(**A**) Receiver-operator characteristic (ROC) curve of the predicted probability of detecting cancers of Gleason score ≥ 7 on biopsy by urinary PSA-PhoSL (solid curve) and urinary PSA-AAL (dashed curve). (**B**) ROC curve of the predicted probability of detecting cancers of Gleason score ≥ 7 on biopsy by the optimum logistic regression model (solid curve), serum PSA (dotted curve), and PSA density (dashed curve).

Next, we determined whether the urinary PSA-AAL and/or urinary PSA-PhoSL levels could predict prostate cancers of GS ≥ 7, which would require definitive treatment. Univariate logistic regression analysis showed that the PSA density (PSAD), urinary PSA-AAL, and urinary PSA-PhoSL levels were good predictors of cancers of GS ≥ 7 (*P* < 0.05), whereas age, serum PSA, prostate volume, and urinary PSA levels were not (Table 2). Stepwise multiple logistic regression analysis showed that PSAD, decreased urinary PSA-AAL, and decreased urinary PSA-PhoSL levels were significant predictors of GS ≥ 7 (*P* < 0.05) in biopsy, whereas the other parameters were not (Table 2). The area under the receiver-operator characteristic curve (AUC) values for the prediction of cancers of GS ≥ 7 by urinary PSA-AAL and urinary PSA-PhoSL levels were 0.69 (95% confidential interval [CI] = 0.56–0.81, *P* = 0.0064) and 0.72 (95% CI = 0.60–0.84, *P* = 0.0014), respectively (Figure 3A). In contrast, the AUC value was 0.59 for serum PSA (95% CI = 0.45–0.73, *P* = 0.17), 0.58 for urinary PSA (95% CI = 0.44–0.72, *P* = 0.23), and 0.63 for PSAD (95% CI = 0.50–0.77, *P* = 0.047). Using urinary PSA-AAL and urinary PSA-PhoSL levels as markers, the AUC values for the prediction of prostate cancer with a GS ≥ 6 were 0.68 (95% CI = 0.54–0.81, *P* = 0.017) and 0.79 (95% CI = 0.67–0.90, *P* < 0.001), respectively, whereas those for the prediction of cancers with a GS ≥ 8 were 0.69 (95% CI = 0.66–0.92, *P* = 0.0010) and 0.77 (95% CI = 0.62–0.91, *P* = 0.0025), respectively. The optimum logistic regression model to predict the probability of detecting cancers with a GS ≥ 7 in biopsy was obtained as *P* = [1 + exp (1.247 + 4.56 × PSAD – 0.00448 × PSA-AAL – 0.0493 × PSA-PhoSL)]^−1^. Using this model, the AUC value for the prediction was 0.82 (95% CI = 0.72–0.92, *P* < 0.0001) (Figure 3B). The sensitivity and specificity of the model at the best cutoff value were 74.1% and 81.5%, respectively. The sensitivity and specificity of each Fuc-PSA are shown in Table 3. The possibility of prostate cancer with a GS ≥ 7 was 52.6% when the PSAD value was > 0.25 in this cohort. The addition of urinary PSA-PhoSL levels of < 31.8 U/mL improved the prediction to 78.9%. The possibility of cancer was only 21.4% when the PSAD value was < 0.25 and the urinary PSA-PhoSL level was > 31.8 U/mL.

**Table 2:** Stepwise logistic regression analysis of variables associated with GS ≥ 7 in biopsy

Variable included	Univariate			Multivariate		
	Odds ratio	95% CI	*p*-value	Odds ratio	95% CI	*p*-value
Age	1.04	0.97–1.13	0.19	–		
PSA	1.07	0.97–1.19	0.12	–		
Prostate volume	0.98	0.95–1.01	0.41	–		
PSA density	12.2	1.18–278	0.031	96.4	3.08–6152	0.004
Urine PSA	0.99	0.98–1.002	0.19	–		
Urine PSA-AAL (× 100 U/ml)	0.61	0.40–0.88	0.0073	0.63	0.39–0.96	0.030
Urine PSA-PhoSL (× 10 U/ml)	0.61	0.41–0.83	0.0006	0.61	0.41–0.83	0.001

**Table 3:** Urinary fucosylated PSAs as markers for prostate cancer and high Gleason score prostate cancer

		Detection for prostate cancer (GS ≥ 6)			
Urinary PSA-AAL cutoff (× 100 U/ml)	Sensitivity		Specificity	
	%	95% CI	%	95% CI
1.97	53.1	38.3–67.5	80.0	56.3–94.3
2.77	69.4	54.6–81.8	60.0	36.1–80.9
3.14	79.6	65.6–89.8	50.0	27.2–72.8
**Urinary PSA-PhoSL cutoff (× 10 U/ml)**	**Sensitivity**		**Specificity**	
	**%**	**95% CI**	**%**	**95% CI**
2.90	54.2	39.2–68.6	85.0	62.1–96.8
3.48	77.0	62.7–88.0	80.0	56.3–94.3
4.54	85.4	72.2–93.9	50.0	27.2–72.8
		**Detection for high Gleason score prostate cancer (GS ≥ 7)**			
**Urinary PSA-AAL cutoff (× 100 U/ml)**	**Sensitivity**		**Specificity**	
	**%**	**95% CI**	**%**	**95% CI**
2.20	71.0	52.0–85.8	63.2	46.0–78.2
2.88	80.6	62.5–92.6	50.0	33.4–66.6
3.09	90.3	74.3–98.0	47.3	30.9–64.2
**Urinary PSA-PhoSL cutoff (× 10 U/ml)**	**Sensitivity**		**Specificity**	
	**%**	**95% CI**	**%**	**95% CI**
3.18	70.0	50.6–85.3	63.2	50.0–78.2
3.49	80.0	61.4–92.3	55.3	38.3–71.4
4.34	90.0	73.4–97.9	39.5	24.0–56.6

## DISCUSSION

In this study, the urinary Fuc-PSA level was found to be associated with the GS in prostate biopsy as well as the presence of prostate cancer in biopsy. The urinary Fuc-PSA level decreased as the GS increased. This non-invasive urinary test could help patients with abnormal PSA levels in deciding whether to undergo the invasive prostate needle biopsy. Urinary Fuc-PSA levels might also be useful in monitoring the status of a patient's prostate cancer.

Several new biomarkers associated with high GS prostate cancer were recently reported. Li et al. recently reported that serum Fuc-PSA could differentiate aggressive prostate cancers from non-aggressive ones [9]. Their method was based on the magnetic bead-based immunoassay, wherein serum total PSA was captured and Fuc-PSA was detected using AAL [9]. Our results were consistent with theirs, although two types of urinary Fuc-PSA were measured in our study. Lectin-antibody ELISA for serum samples exhibits poor sensitivity and specificity, due to numerous serum proteins interfering with the specific binding of lectin to Fuc-PSA. Compared with serum, urine contains much less proteins and the PSA levels are higher by a > 2-fold magnitude. Our newly developed lectin-antibody ELISA could detect urinary Fuc-PSA.

There are 11 different fucosyltransferases (Fut), classified into four groups. Fut1 and 2 are involved in the synthesis of α1-2 fucose (H type), Fut3, 4, 5, 6, 7, and 9 are involved in the synthesis of α1-3/α1-4 fucose (Lewis type), and Fut8 is involved in the synthesis of α1-6 fucose (core type) [2]. In the prostate, only Fut8 is expressed by the prostate cancer cell lines PC3 and DU145 [6]. Normal prostate epithelial cells (PrECs) express Fut3 and Fut8, and normal prostate stromal cells (PrSCs) express Fut8 (unpublished data). Core-type Fuc-PSAs are produced from both normal PrECs and prostate cancer cells, whereas Lewis-type Fuc-PSAs originate only from normal PrECs. AAL binds to both Lewis-type and core-type fucosylated glycoproteins, whereas PhoSL binds to core-type fucosylated glycoproteins only. Although the urinary PSA-AAL levels were significantly correlated with urinary PSA-PhoSL levels (Spearman's *r* = 0.477), multivariate analysis showed that both PSA-AAL and PSA-PhoSL remained significantly associated with high GS prostate cancer in biopsy. The combination of both types of urinary Fuc-PSA levels resulted in a higher sensitivity and specificity for the detection of high-risk prostate cancer (GS ≥ 8) in biopsy.

There were several limitations encountered in this study. The number of patients with GS 8–9 in this cohort was also high compared with the normal distributions in the USA. Given that this was a pilot study with a small population size, further large-scale and multi-institutional studies are warranted to confirm these findings. In the USA, many cases of newly diagnosed prostate cancer are followed by active surveillance, who have only a small volume of cancerous cells. Urinary Fuc-PSA tests should also be performed on the patients under active surveillance to discriminate those with high GS prostate cancer who will require definitive therapy. The mechanisms of decreased urinary Fuc-PSA levels in patients with high GS prostate cancer are speculative. In the normal prostate, luminal cells secrete PSA into the lumen, resulting in high PSA levels in the urine. It is well known that urinary PSA is not a good marker for prostate cancer, in contrast to serum PSA [10]. In the liver, fucosylation is a signal for the secretion of glycoproteins into bile ducts. A disruption of this system causes decreased levels of fucosylated glycoproteins in bile [11]. The disturbance of PSA fucosylation in high GS prostate cancers might lead to the decrease in Fuc-PSA secretion into the prostatic fluids. Another possible mechanism of decreased urinary Fuc-PSA in men with high GS prostate cancer is “field cancerization” or “field effect.” Prostate lesions develop in a multifocal pattern and the normal prostate epithelial adjacent to prostate cancer are found to be morphologically and genetically distinct from cells of distant tissue [12]. In the presence of high GS prostate cancer, the fucosylation in healthy prostate cells might have been changed, resulting in the decreased levels of secreted Fuc-PSA in the prostatic fluid. The detailed mechanism of how Fuc-PSAs are produced should also be studied further.

In conclusion, urinary levels of Fuc-PSA were decreased in men with prostate cancer compared with their cancer-free counterparts, and were significantly associated with a high GS in prostate biopsy outcomes. The specific finding of a decreased urinary Fuc-PSA level might be useful for recommending prostate biopsy to patients with elevated PSA. Further large-scale and multi-institutional studies are necessary to validate these initial findings.

## MATERIALS AND METHODS

### Sample collection

Urine samples were collected from Osaka University Hospital. Approval and written informed consents were obtained from our Institutional Review Board and patients, respectively, before initiating the study. All patients were subjected to DRE, wherein three finger strokes were performed per prostate lobe. Immediately after DRE and before prostate biopsy, first voided urine samples (40–60 mL) were collected from 69 patients. Samples were initially stored at 4°C for up to 6 h prior to aliquoting and then transferred to −80°C until analysis. Patients received a transrectal ultrasound-guided 12-core biopsy. If patients had suspicious lesions on their magnetic resonance images, an additional targeted biopsy was performed. The percentage of positive cores was calculated as the positive core number divided by the total core number. The percentage of prostate cancer core length was calculated as the sum of each percentage of prostate cancer core length divided by the total core number.

### Lectin-antibody ELISA for PSA-AAL and PSA-PhoSL quantification

Lectin-antibody ELISA was performed as previously described [6, 13, 14]. Ninety-six-well ELISA plates were coated with anti-human-free PSA IgG (Dako, Carpinteria, CA, USA) pre-incubated with 10 mM sodium peroxidase. The coated plates were blocked for 1 h with phosphate-buffered saline (PBS) containing 3% bovine serum albumin and then washed with PBS containing 0.1% Tween 20. A 50 μL aliquot of the urine was placed in each well and the plates were incubated for 1 h at room temperature. Diluted biotinylated AAL and diluted biotinylated PhoSL (1/1,000) were placed in each well for the detection of AAL-PSA and PhoSL-PSA, respectively, and were allowed to react at room temperature for 1 h. Peroxidase-conjugated avidin was added to each well and incubation was carried out at room temperature for 1 h followed by 15-min incubation with tetramethylbenzidine. To stop the reaction, 1N sulfuric acid was added to each well.

### Statistical analyses

Results were expressed as a median (range). Statistical analyses were done using GraphPad Prism 5.0 for Windows. Mann-Whitney tests were used to analyze the difference between two categories, and stepwise associations between pathological findings of biopsy specimens (negative, or adenocarcinomas with Gleason scores of 6, 7, or 8–9). Other clinical characteristics were compared using the Jonckheere-Terpstra test. Correlation analysis was done using Spearman analysis. Significant factors predicting GS ≥ 7 were identified by stepwise logistic regression analysis. Variables entered into the model were the patient's age, and PSA, prostate volume, PSA-AAL, and PSA-PhoSL levels. All *P* values were two-sided, with statistical significance being accepted at *P* < 0.05. All statistical analyses were performed using SPSS version 11.0.1 (SPSS, Chicago, IL, USA), GraphPad Prism 5 (GraphPad Software, La Jolla, CA, USA), and R version 2.13.0 with the RcmdrPlugin. EZR package (Saitama Medical Center, Jichi Medical University, Japan), which is a graphical user interface for R (The R Foundation for Statistical Computing).

## Abbreviations

PCa: prostate cancer
PSA: prostate-specific antigen
AAL: Aleuria aurantia lectin
PhoSL: Pholiota squarrosa lectin
Fuc-PSA: fucosylated prostate-specific antigen
GS: Gleason score.
